# 

**Published:** 1835-04-01

**Authors:** 


### BIBLIOGRAPHICAL RECORD;

OR,

*Works received for Review since last Quarter.*

1. The Practice of the Liverpool Ophthalmic Infirmary, for the Year 1834; being the first Special Report. By Hugh Neill, Surgeon to the Charity. Octavo, pp. 54, with Plate.

---

2. An Exposition of the Nature and Treatment of Continued Fever. By Henry M'Cormac, M.D. Octavo, pp. 202. Longman and Co. 1835.

---

3. A Treatise on Rickets; with a new Theory of Ossification, and a Plate and Description of an improved reclining Couch for the Distorted. By Geo. Hume Weatherhead, M.D. &c. Small 8vo, pp. 128. Second Ed. Highley, 1835.

---

4. Observations on the Causes and Treatment of Ulcerous Diseases of the Leg. By J. C. Spender, M.R.C.S. Octavo, pp. 210. Longman and Co. 1835.

---

5. A Series of Anatomical Plates, &c. By Jones Quain, M.D. Fasciculus 22—33. Division I., the Muscles. March. 1835.

☞ *We shall notice these fully in our next.*

---

6. Lectures on the Morbid Anatomy, Nature, and Treatment of Acute and Chronic Diseases, delivered in the Theatre of Anatomy, Webb-st. by the late John Armstrong, M.D. Edited by Joseph Rix, M.R.C.S. Octavo, pp. 851. Baldwin and Co. Paternoster-row. April, 1834.

☞ *By some accident, this valuable copy of Dr. Armstrong's Lectures was omitted in our Bibliographical Record. Mr. Rix, a very talented young surgeon, was induced during several courses to take careful notes of Dr. Armstrong's lectures—so careful, copious, and exact were they, that the late lamented lecturer solicited the loan of them from the editor, during his illness, that they might be read to his pupils, when he was unable to deliver them* viva voce. *This fact is attested by Dr. Armstrong himself, in a letter to Mr. Rix, and after such a testimony in favour of the correctness, fidelity, and amendments of the lectures, it would be folly to add a single word by way of recommendation.*

7. The Principles of Physiology, applied to the Preservation of Health, and the Improvement of Physical and Mental Education. By A. Combe, M.D. Third Edition, revised and enlarged, 1835.

☞ *The fact of a work coming to a third edition in eleven months speaks volumes.*

---

8. The Nature of Cholera investigated. By John George French, M.R.C.S. &c. Octavo, pp. 54. Rivington's, Feb. 1835.

---

9. A Treatise on Insanity, and other Disorders affecting the Mind. By James Cowles Prichard, M.D. Senior Physician to the Bristol Infirmary. Octavo, pp. 483. Sherwood and Co. March, 1835.

---

10. The Epidemics of the Middle Ages; from the German of J. F. C. Hecker, M.D. Translated by B. G. Babington, MD. FRS. Small 8vo, pp. 195. Sherwood and Co. March, 1835.

---

11. Outlines of Comparative Anatomy. By Robert E. Grant, M.D. Professor of Comparative Anatomy and Zoology in the University of London, &c. *Part the First*, containing Osteology, Ligaments, and Muscles, illustrated with 65 Wood-cuts. Octavo, pp. 144. Bailliere, Regent-street, March 1, 1835. Price seven shillings.

☞ *As this estimable Work is now publishing in parts, and at a cheap rate, we strongly recommend it to all those who wish to take a comprehensive view of comparative anatomy.*

---

12. Descriptive Catalogue of the Preparations in the Museum of the Royal College of Surgeons in Ireland. By John Houston, M.D. &c. Octavo, pp. 248. Dublin, 1835.

☞ *A valuable vade-mecum for the professional visitor of the Museum.*

---

13. Cyclopædia of Practical Medicine, Part XXV. containing articles on Worms, Wounds, Yaws, Constipation, Hæmorrhoids, Liver-Diseases, Porrigo, Prurigo,

Rupture of Heart, Diseases of Spinal Marrow, Stomach, Tetanus, Med. Bibliography.

☞ *There is but one more Part to conclude this national and highly valuable publication.*

---

14. Pathological Researches on Phthisis. By E. Ch. A. Louis, M.D. Physician to La Pitié in Paris. Translated from the French, with Introduction, Notes, Additions, and an Essay on Treatment. By Charles Cowan, M.D. &c. Octavo, pp. 388. E. Portwine, London, March, 1835.

---

15. De l'Emploi de l'Excision, et de la Cauterization, à l'Aide du Nitrate de l'Argent, fondu dans l'Ophthalmie Blennorrhagique, &c. Par E. T. Julliard, de Geneve, Quarto, pp. 88. Paris, 1835.

---

16. Principles and Practice of Obstetric Medicine, &c. By Dr. Davis. Part 40. March, 1835.

---

17. Lectures on the Means of promoting and preserving Health, delivered at the Mechanic's Institution, Spitalfields. By T. Hodgkin, M.D. Small 8vo, pp. 449. March, 1835.

☞ *These lectures are exceedingly well adapted for the non-professional reader, especially for those of the working-classes. They are by no means unworthy of professional perusal also.*

---

18. The Phrenological Journal and Miscellany, No. 43. March 1, 1835.

---

19. A Compendium of the Diseases of the Skin, with Cases; including a particular Consideration of the more frequent and intractable forms of these Affections. By Jonathan Green, M.D. M.R.C.S. Octavo, pp. 371. Whittaker, London, March, 1835.

---

20. The Cyclopædia of Practical Medicine and Surgery—a Digest of Medical Literature. Edited by Isaac Hays, M.D. Part I. Philadelphia, July, 1833. Octavo, pp. 108, double columns.

☞ *It is expected that this work will be comprised in eight volumes, being compiled from the various dictionaries of medicine, English and foreign.*

---

21. Anatomical Description of the Parts concerned in Inguinal and Femoral Hernia. Translated from the French of M. Jules Cloquet, with Lithographic Plates, &c. By A. Melville M'Whinnie, Assistant-Teacher of Practical Anatomy, at St. Bartholomew's Hospital. Octavo, pp. 50. Highley, Dec. 1834.

---

22. An Essay on the Use of the Liquor Potassæ and Liquor Alkalinus in the Treatment of Malignant Cholera. By Henry W. Dodd, M.R.C.S. Durham, 1834. Pp. 33.

---

23. The Anatomy, Physiology, and Diseases of the Teeth. By Thos. Bell, F.R.S. &c. Second Edition, Dec. 1834. Highley.

---

24. On the Preparation and Medicinal Employment of Aconitine, by the Endermic Method, in the Treatment of Tic Douloureux and other Painful Affections. By Alex. Turnbull, M.D. Octavo, pp. 48. Longman and Co.

---

25. A familiar Description of the Nature, Symptoms, and ordinary Modes of Treatment of Cataract, &c. By John Stevenson, Esq. &c. 1834.

---

26. The Pathology and Diagnosis of the Chest; illustrated by a Rational Exposition of their Physical Signs. By Charles J. B. Williams, M.D. Third Edition, with important Additions, and with new Researches on the Sounds of the Heart.

---

27. The Marriage Almanack; or Ladies' Perpetual Calendar, in which every Day of the Year is marked with Reference to its important Epochs. Translated from the French of Dr. Desberger, by an English Physician. 12mo. Schloss, April, 1835.

---

28. A brief Account of the Origin and Progress of the Patent Syringe, or Stomach Pump, &c. By John Read, Regent Circus, Piccadilly. Octavo, pp. 79. March, 1835.

☞ *We have also received from Mr. Read a small instrument, "on his new patent principle, with flexible rectum-tube, &c." which we understand has been highly approved by Sir B. Brodie, Mr. Guthrie, and Mr. Earle. It appears to be very perfect and ingenious in its mechanism.*

---

29. Illustrations of the Elementary Forms of Disease. By Robert Carsewell, M.D. Fasciculus VII.—Mortification.

---

30. Fasciculus 24 of Dr. Quain's Anatomical Plates is just received.

INDIGESTION—CHANGE OF AIR—TOUR OF HEALTH.

AN ESSAY on INDIGESTION, or MORBID SENSIBILITY of the STOMACH and BOWELS, as the Source of various Diseases, mental and corporeal. By JAMES JOHNSON, M.D. Physician Extraordinary to the KING. *Eighth Edition, price 6s. 6d.*

BY THE SAME AUTHOR,

2. CHANGE of AIR; or PURSUIT of HEALTH, through France, Switzerland, and Italy. *New Edition, greatly enlarged, price 8s. 6d.*

(Sequel to "CHANGE OF AIR.")

3. THE RECESS; a Tour of Health and Pleasure to the Highlands and Islands. *Price 7s. 6d.* Highley, 32, Fleet Street.

**CRITICAL NOTICES.**

"Of all the popular Tours of which British literature has recently been so prolific, this is immeasurably the best. To attempt an analysis of a work embracing such a treasure of anecdote and instruction, would be an idle task There is no class of general readers which may not derive pleasure and profit from the perusal of this volume."—BALLOT.

"Dr. JOHNSON is a vigorous and independent thinker, while his opinions are slightly tinctured with cynicism, which gives them an agreeable relish. His style is clear, bold, and expressive; so that when he least aims at effect, he leaves a more vivid impression on us of the object of his reflections, than others would by an elaborate description."—MORNING HERALD.

"The Author, out of his abundant stock of knowledge and reflection, has constructed a volume with which all classes will be pleased."—ATLAS.

"The medical portion embraces numerous remarks which we would recommend all invalids and their medical advisers to peruse, before they decide upon the dangerous experiment of foreign travel."—SPECTATOR.

"In his description of the different countries he passed through, and the many objects of curiosity that attracted his notice, Dr. JOHNSON has preserved a freshness and originality that reflect high credit upon his talent, as a writer and acute observer."—LONDON MED. & PHYS. JOURN.

"The present publicatian is the most entertaining and edifying that has issued from the press for many years."—GAZETTE OF HEALTH.

THE RECESS.

"I must say I never saw more various—I might add, profound research so pleasantly seasoned with satire."—AUTHOR OF TREMAINE.

### INDEX.

A.

Acarus scabiei, report on the ...... 507
Aconitine, Dr. Turnbull on ........ 434
Aconitine, preparation of .......... 435
Algiers, medical statistics of........ 509
Amaurosis, treated by strychnine.... 560
Amenorrhœa, report on, by Dr. Carbutt ........................ 255
Amenorrhœa, treatment of, by irritation of the mammæ............ 511
Anatomical pathology of the nervus vagus ...................... 170
Anatomy, Mr. Quain's treatise on ... 137
Anatomy of the lymphatic system .. 220
Anatomy bill, complaints against the, 588
Andral, M. his clinique médicale .... 398
Aneurism by anastomosis, treated by caustic ..................... 512
Animation, suspended, recovery from, 434
Annesley, Mr. on dysentery ........ 425
Antimony after wounds, &c......... 193
Arachnoid, diseases of the .......... 402
Armstrong, Dr. memoir of his life, by Dr. Boott...................... 69
Arsenic, detected after 14 months' interment ................... 464
Arteries, thesis on the different modes of obliterating.................. 89
Assistants to hospitals, plurality of .. 168
Austrian system of medical education.. 272

B.

Beaumont, Dr. his experiments on the gastric juice ...................... 49
Bellingeri on facial neuralgia........ 225
Biceps flexor cubiti, rupture of tendon, 432
Birmingham school of medicine .... 581
Bladder, Mr. Guthrie on diseases of the 107
Bladder, anatomical description of the, 108
Bladder, disease of its "elastic structure" ......................... 111
Bladder, pouches in the............ 111
Bladder, "fluttering blow of the" ... 112
Bladder, chronic thickening of the .. 135
Bladder, Mr. Crosse on calculi in the, 321
Bladder, chronic inflammation of the, 322
Bladder, sacculi of the ............ 323
Bladder, disease of kidneys from stone, 325
Bladder, on sounding for stone in the, 326
Blood, Mr. Thackrah on the ........ 368
Blood, size of its globules .......... 371
Blood, components of.............. 371
Blood, coagulation of the .......... 373
Blood, arterial and venous.......... 376
Blood, affected by the states of the animal system ................. 377
Blood, milky appearance of ........ 378
Blood, state of, in its vessels........ 379
Blood, states of, in disease..... .... 380
Blood, purulent alteration of the .... 500
Blundell's, Dr. practice of obstetricy.. 73
Blundell, Dr. on hidriotic fever...... 75
Boott, Dr. his life of Dr. Armstrong.. 69
Boott, Dr. on marsh fever.......... 69
Bostock, Dr. on the pathology of the urine ...................... 454
Botany, Mr. Burnett's outlines of.... 436
Bouillaud's refutation of Majendie's theory of the sounds of the heart .... 231
Brain, functional disturbances from lesions of the.................. 410
Brodie, Sir B. on hæmorrhoids...... 564
Brodie, Sir B. on prolapsus recti .... 571
Brodie, Sir B. on excrescences of the rectum........................ 573
Broussaisism, strictures on ........ 209

C.

Calculi, chemical composition of .... 308
Calculi, growth of ................ 309
Calculi in the kidneys and ureters.... 312
Calculi in the urethra ............ 316
Calculi in the prostate ............ 318
Calculi in the bladder.............. 321
Calculi removed by the urethra...... 332
Calculi, small and large, symptoms of, 332
Calculi, *mode* of removing per urethram 333
Calculous complaints in India ...... 187
Calculous diseases, causes of........ 306
Calculus, Mr. Crosse's treatise on the, 305
Carbutt, Dr. his clinical report..... 246
Carditis, cephalitis, case of ........ 153
Cataract, memoranda on .......... 542
Caustic for aneurism by anastomosis.. 512
Cervical vertebræ, remarkable distortion of........................ 557
Chloruret, for the removal of putrid corpses ...................... 526
Cholera, Dr. Hawthorne on ........ 156
Cholera, Mr. George on............ 483
Cholera, souvenirs du, a poem ...... 535
Chorea, Dr. Carbutt's report on .... 246
Chyme, Dr. Beaumont's description of, 66
Clinique médicale, M. Andral's...... 398

Circulation, Harvey *said* not to have discovered .................... 31
Cloquet, M. Jules, his description of the anatomy of hernia .......... 382
Concours, for the clinical chair of surgery........................ 79
Concours, thesis on lithotomy and lithotrity ...................... 79
Concours, thesis on hæmorrhoids and prolapsus recti ........... ..... 89
Concours, thesis on the methods of obliterating arteries ............ 89
Concours, appeal of French surgeons against its violation ............ 533
Conjunctiva, sensibility of the ...... 562
Convulsions, tetano-epileptic, case of, 449
Corrosive sublimate, poisoning by ... 515
Crepitatio musculorum, Dr. Johnson's case......................... 168
Crosse, Mr. on the urinary calculus.. 305
Crural canal, anatomy of .......... 393
Cutaneous diseases; Willan's arrangement best .... ............... 206

D.

Dead, the mighty ................ 161
Death from hanging, observations on, 518
Death, difficulty of determining its causes ......................... 519
Deaths of kings, Sir H. Halford on the, 585
Delirium, as a symptom, M. Andral on 415
Dentology, Mr. Bell on ............ 487
Dentology; a poem! by Solyman Brown, A.M. ................... 487
Diaphragm, laceration of the—legal question ...................... 529
Digestion, solution of the food during 60
Digestion, time required for ........ 62
Digestion, muscular actions of the stomach during ........... .... 64
Digitalis used endermically.......... 207
Dislocation of Femur, proposed in morbus coxæ .................. 183
Ductus choledochus, obstruction of the 187
Dupuytren, M. on fractures of the carpal end of the radius........... 216
Dura mater, diseases of the ........ 399
Dura mater, lesions of the ........ 407
Dysentery, endemic .............. 154
Dysentery, Mr. Annesley on........ 425
Dysentery, treatment of............. 428

E.

Ear, pendulous tumor from the .... 186
Edwards, Dr. on the physiological characters of the races of mankind.. 189
Elbow-joint, Mr. Guthrie on injuries of the ........................ 257
Endemic dysentery................. 154
Endermic use of digitalis .......... 207
Endermic medication, Dr. Sigmond on 492
Enteritis, Dr. Griffin on .......... 477
Enteritis, purging in .............. 479
Epidermis of infants, exfoliation of the 541
Erysipelas, Dr. M'Dowel on ........ 162
Eye, Mr. Wardrop's morbid anatomy of the ........................ 140

F.

Fees, a legal claim in France........ 538
Fibrous tumors of the uterus, Messrs. Dupuyten, Boivin, and Dugés on .. 34
Fibrous tumors of uterus, varieties of, 35
Fibrous tumors of uterus, symptoms of 44
Fistula lacrymalis, catgut bougie for.. 564
Fracture, ununited, cured by friction, 177
Fracture, compound, of tibia ...... 243
Fractured ribs, cases of............ 241
Fractured femur—no union ........ 242
Frottement in peritonitis .......... 160

G.

Gastric juice, Dr. Beaumont's experiments on the .................. 49
Gastric juice, extraction of for experiment .......................... 51
Gastric juice, properties of the...... 51
Gastric juice, composition of........ 52
Gastric juice, solvent power of...... 52
Gastric juice, Tiedeman and Gmelin on 53
Gastric juice; do other fluids exert its solvent power.................. 55
Gastric juice, its action in digestion.. 57
Germany, midwifery in ............ 193
Germany, sarsaparilla in .......... 194
Germany, medical literature of...... 194
Gout, Dr. Leese on ............... 151
Graves, Dr. his introductory lecture.. 583
Green, Dr. on the non-mercurial treatment of syphilis ................ 265
Griffin, Messrs., on affections of the nerves ......................... 1
Griffin, Dr. on enteritis ............ 477
Gums, Mr. Waite on the .......... 474
Guthrie, Mr. on diseases of the bladder 107

H.

Hæmorrhage after lithotomy........ 342
Hæmorrhage from the internal pudic, 346
Hæmorrhage, deep, after lithotomy .. 347
Hæmorrhoids and prolapsus recti.... 204
Hæmorrhoids, Sir B. Brodie on...... 564
Hæmorrhoids, nature of............ 564
Hæmorrhoids, abscess connected with 565
Hæmorrhoids, protrusion of ........ 566
Hæmorrhoids, treatment of ........ 567
Hæmorrhoids, operation for ........ 568
Halford, Sir Henry, on the deaths of kings ......................... 585
Harvey, his claims to the discovery of the circulation denied ........ 31
Hawkins, Mr. on injuries of the nerves 234

Hawthorne, Dr. on cholera ........ 156
Head, injuries of, followed by mania, 256
Head, injuries of, Mr. Guthrie on ... 257
Head, injuries of, report on ........ 259
Health, Dr. Smith's philosophy of ... 421
Health, public, on agencies affecting, 524
Heart, malignant tumors of the .... 357
Heart, diseased, case of............ 461
Hemicrania, case of ............... 430
Hemiplegia in pregnancy .......... 152
Hepatized spleen, case of .......... 272
Hernia, M. Cloquet's anatomy of .... 383
Hernia, inguinal, anatomy of ....... 383
Hernia, femoral, anatomy of........ 392
Hernia, anatomy of the epigastric and obturatrix arteries in relation to .. 396
Hidrosis, Dr. Blundell on .......... 75
Hints on sloughing fauces.......... 145
Hints on diabetes ................ 145
Hints, miraculous ................ 146
Hip diseased, femur proposed to be dislocated in ................... 183
Hospital for lithotrity ............. 589
Hunger, theory of ................ 68
Hydatids, formation of............ 431
Hydrocephalus, case of ............ 430
Hydro-ferro-cyanate of quinine ..... 211
Hydrophobia, Mr. Pettigrew on .... 180
Hypertrophy of the mammæ........ 179
Hysteria, epileptic, curious case of .. 229

I.

Impotence, anecdotes of ........... 523

Infanticide suspected — medico-legal consultation .................. 527

Infants, peculiar affection of lungs in, 460

Infants, exfoliation of the epidermis of 541

Influenza in Edinburgh ............ 432

Ingleby, Dr. on the signs of pregnancy 468

Inguinal canal, anatomy of the...... 389

Injuries of the nerves, Mr. Hawkins on 234

Injuries of the head, report on ...... 259

Insanity, Dr. Prichard on .......... 442

Insanity, varieties of .............. 442

Insanity; monomania ............ 444

Insanity; mania.................. 445

Insanity; dementia ............... 446

Insanity, terminations of .......... 447

Irritation of the spinal cord, Mr. Stanley on .................... 353

Itch-insect, Institute report on the .. 507

J.

Jaundice, cases of, by Dr. Carbutt .. 248

Johnson, Dr. his case of gastric neuralgia ...................... 166

Johnson, Dr. his case of crepitatio musculorum .................. 168

Johnson, Dr. on the Baths of Pfeffers, 281

Johnson, Dr. his case of tetano-epileptic convulsions ................ 449

Johnson, Dr. on acute and chronic rheumatism.................. 460

Johnson, Dr. his cases of diseased kidneys ...................... 484

Johnson, Mr. H. J. on venereal ulcers of the rectum ................. 574

K.

Kidneys, on calculi in the .......... 312
Kidneys, diseased, in connexion with irritation of the spinal cord ...... 353
Kidneys, some cases of disease of the, 484
Kidneys, disease of, marked by symptoms of disease in the bladder .... 484

L.

Labour, difficult, from malposition of head......................... 173
Labour, varices of vagina ruptured in, 224
Laceration of the diaphragm—legal question ..................... 529
Latham, Mr. on uterine hæmorrhage, 150
Lee, Dr. on the pathology of the uterus ....................... 364
Liquor potassæ in Cholera.......... 483
Liston, Mr. on diseases of the rectum, 489
Litho-cystotomy, Mr. Crosse on .... 336
Litho-cystotomy, mode of performing, 337
Litho-cystotomy, treatment after.... 339
Litho-cystotomy, inflamed bladder after 340
Litho-cystotomy, diffuse cellular inflammation after ............... 340
Litho-cystotomy, nervous exhaustion after ........................ 340
Litho-cystotomy, perineal fistula after, 341
Litho-cystotomy, wound of rectum in, 342
Lithotomy and lithotrity, Concours Thesis on...................... 79
Lithotomy, causes of death after .... 113
Lithotomy in India................ 187
Lithotomy in Italy................ 196
Lithotomy and lithotrity, comparative cases of..................... 196
Lithotomy, hæmorrhage after ...... 342
Lithotomy, tables shewing the results, 349
Lithotrity, death from ............. 199
Lithotrity, Mr. Crosse on .......... 335
Liverpool Ophthalmic Infirmary, report of......................... 559
Lunatic asylum in Germany ........ 476
Lungs, peculiar affection of in infants, 460
Lymphatic system, anatomy of the .. 220

M.

M'Cormac, Dr. on fever ........... 496
M'Dowel, Dr. on erysipelas ........ 162
Macfarlane, Dr. his report on tumors, 545
Mammæ, hypertrophy of the........ 179
Mammæ, irritation of, for amenorrhœa 511
Man, physiological characters of his races ......................... 189

Maniacal symptoms after injuries of the head .......................... 256
Marsh fever, Dr. Boott on.......... 69
Mayo, Mr. his introductory lecture.. 141
Medical education in Austria ...... 272
Medical opinion, state of in Paris.... 497
Medical men, responsibility of, in France .......................... 536
Medical men; fees a legal claim in France .......................... 538
Medical superstitions in France...... 543
Medico-chirurgical Transactions of Edinburgh. Fas. II............. 429
Melanosis of the eye-ball .......... 221
Mercurial and non-mercurial treatment of syphilis .................. 264
Miasmata, matter detected in ...... 223
Midwifery, wretched in Germany.... 193
Montgomery, Dr. on difficult labour.. 173
Morbid anatomy of the eye, Mr. Wardrop's .................... 140
Murder from monomania .......... 520
Murder detected from the skeleton, 12 years after death ............ 521
Muscles of the shoulder, partially ossified in young recruits .......... 514

N.

Nephritis, acute, case of .......... 452
Nerves, Messrs. Griffin on affections of the .......................... 1
Nerves, Mr. Hawkins on injuries of the 234
Neuralgia, gastrica, Dr. Johnson's case 166
Neuralgia, facial, Bellingheri on .... 225
Neuralgia of leg and foot—sciatic nerve cut ...................... 229
Neuralgia, case of ................ 244

O.

Obstetricy, Dr. Blundell's practice of, 73
Orbital linings, case of inflammation of 430
Ossification of the deltoid..... .... 514
Outlines of botany, Mr. G. Burnett's, 436

P.

Paris, state of medical opinion in.... 497
Paternity, French laws on.......... 524
Peritonitis, Trottement............ 160
Pettigrew, Mr. on hydrophobia...... 180
Pfeffers, Dr. Johnson on the baths of.. 281
Phlebitis, from tying varicose veins .. 264
Phlebitis, traumatic, cases of........ 503
Phlegmasia dolens, case of.......... 173
Philosophy of health, Dr. Smith's .. 421
Phrenology, human and comparative, 221
Phthisis mistaken for diseased heart.. 171
Phthisis, incipient, relieved ........ 177
Pia mater, diseases of the.......... 402
Pia mater, lesions of the .......... 408
Pleasure, the end of existence ...... 423
Pleuro-pneumonia, case of ........ 155
Pneumo-gâstric nerve, pathology of.. 170
Pneumo-gastric nerve, pathology of.. 218
Poisoning by arsenic, detection of, after 14 months ...................... 464
Poisoning by corrosive sublimate.... 515
Poisoning detected 7 years after death, 523
Poisoning with belladonna ........ 528
Polypi of the uterus, M.M. Dupuytren, Boivin and Dugés on ............ 34
Polypi of uterus, description of .... 35
Polypi of uterus, terminations of.... 38
Polypi of uterus, causes of ........ 40
Polypi of uterus, examination for.. 42
Polypi of uterus, distinguished from inversion of the uterus .......... 45
Polypi of uterus, M. Dupuytren's treatment of ...................... 46
Polypus of the bladder simulating stone ...................... 329
Population returns — influence of crowding........................ 582
Practical hints, Dr. Peacock's ...... 145
Pregnancy, signs of .............. 468
Pregnancy, vomiting in............ 469
Pregnancy, sanguineous discharges in 469
Pregnancy, state of the breasts in .. 469
Pregnancy, fætor of discharges in.... 469
Pregnancy, state of os uteri in...... 470
Pregnancy, state of hypogastrium in.. 471
Pregnancy, obliquity of uterus in.... 472
Pregnancy, fœtal movements in.... 473
Pregnancy, auscultation during...... 474
Prichard, Dr. on insanity .......... 442
Prolapsus recti and hæmorrhoids .... 204
Prostate gland, abscess of the ...... 133
Prostate gland, enlargement of...... 134
Prostatic calculi, Mr. Crosse on .... 320
Public spirit of the surgeons of Paris, 533
Pudic, internal artery, hæmorrhage from the ...................... 346
Purulent alteration of the blood .... 500
Putrid corpses, removed without danger ......................... 526

Q.

Quain, Mr. his treatise on anatomy.. 137
Quinine, hydro-ferro cyanate of .... 211
Quinine, monstrous doses of........ 245

R.

Radius, M. Dupuytren on fracture of its carpal extremity ............ 216
Rectum, Mr. Liston on disease of the, 489
Rectum, Sir B. Brodie on prolapsus of the .......................... 571
Rectum, Sir B. Brodie on excrescences of the ...................... 573
Rectum, Mr. H. J. Johnson, on venereal ulcers of ................ 574
Responsibility of medical men in France.......................... 536

Retention of urine, operations for.... 128
Rheumatism, cases of, by Dr. Carbutt, 251
Rheumatism, Dr. Johnson on ...... 461
Rheumatism, acute, nature and treatment of .................... 504
Rhinoplastic, performed by Dieffenbach 544
Right-handed; why are we so? .... 587

S.

Sarsaparilla in Germany .......... 194
Salt, Dr. Mateer on the injurious effects of .................... 480
Scarlatina, epidemic in Heriot's hospital....................... 433
Sciential medicine, Mr. Eden's ...... 362
Scott, Mr. on tic douloureux........ 97
Secale cornutum as an emmenagogue, 233
Serum, blue from a blister ........ 213
Sigmond, Dr. on endermic medication 492
Sims, Dr. on malignant tumors of the heart and lungs ................ 357
Smith, Dr. his philosophy of health.. 421
Solanine in potatoes .............. 206
Sounding, Mr. Crosse on ..... .... 326
Sounding, by the catheter.......... 328
Sounding, *rotatory*................ 328
Sounding, dangers of .............. 329
Spinal cord, functional affections of the 3
Spinal cord affections of its cervical portion .................... 9
Spinal cord, affections of the dorsal portion ...................... 19
Spinal cord, affections of the lumbar portion . ................... 20
Spinal cord, general irritation of the, 21
Spinal irritation, resembling inflammation....................... 22
Spinal inflammation, acute ........ 24
Spinal irritation, treatment of ...... 27
Spinal dura mater, Professor Albers on inflammation of the ............ 227
Spinal cord, extravasation of blood on 263
Spinal cord, Mr. Stanley on irritation of the...................... 353
Spine, scrofulous caries of the, curious case of...................... 557
Stomach, state of, during digestion.. 59
Stone, hospital for the ............ 589
Stricture, Mr. Guthrie on .......... 118
Subclavian, case of ligature of the.. 214
Syphilis, on the non-mercurial treatment of ..................... 264

T.

Tetano-epileptic convulsions; curious case ......................... 449
Thackrah, Mr. on the blood ....... 368
Tic douloureux, Mr. Scott on ...... 97
Tic douloureux, progress, &c. of .... 98
Tic douloureux, species of.......... 99
Tic douloureux, causes of .......... 100
Tic douloureux, treatment of ...... 101
Transfusion of blood, successful .... 230
Traumatic phlebitis, cases of........ 503
Truth, triumph of ........... .... 150
Tumors, malignant, Dr. Sims on .... 357
Tumors, Dr. Macfarlane's report on.. 545
Tumors of the head and neck ...... 545
Tumors, adipose and encysted ...... 546
Tumors, malignant................ 549
Tumors of the mamma ............ 551
Tumors, scirrhous and fungous...... 552
Turnbull, Dr. on aconitine.......... 434

U.

Umbilical cord, desiccation of the ... 540
Ununited fracture, cured by friction, &c. 177
Ureters, on calculi in the .......... 312
Urethra, Mr. Guthrie on the........ 115
Urethra, length of ................ 116
Urethra, structure of the .......... 118
Urethra stricture of the............ 119
Urethra, hæmorrhage from the...... 126
Urethra, calculi in the ............ 316
Urinary calculus, Mr. Crosse on the.. 307
Urinary calculus, chemical composition of the .................... 308
Urinary calculus, growth of ........ 309
Urine, operations for retention of.... 128
Urine, Dr. Bostock on the pathology of the ........................ 454
Urine, aqueous . ................ 454
Urine, sub-aqueous .............. 455
Urine, lithic .................... 455
Urine, phosphatic ............... 457
Urine, purpuric ................. 457
Urine, albuminous ............... 458
Urine, saccharine ............... 459
Uterine hæmorrhage, Mr. Latham on, 150
Uterine inflammation; Dr. Churchill's cases ........................ 481
Uterus, MM. Dupuytren, Boivin, and Dugés on polypi of............... 34
Uterus, Dr. Lee on the pathology of the 364
Uterus, inflammation of the . ...... 364
Uterus, fibrous tumors of the ...... 366

V.

Varices of vagina, ruptured in labour, 224
Varicose tumor of scalp............ 212
Venereal ulcers of the rectum, Mr. H. J. Johnson on.................. 574
Venereal ulcers of the rectum, with condylomata ................. 575
Venereal ulcers of the rectum, of phagedenic character ............. 576
Venereal ulcers of the rectum, with ulceration of other mucous membranes ....................... 578
Venereal ulcers of the rectum, with various secondary symptoms...... 579
Vesalius, anecdotes of............ . 213

W.

Waite, Mr. on the gums............ 474
Wardrop, Mr. his morbid anatomy of the eye........................ 140
Williams, Dr. on the treatment of syphilis ........................ 268

